# Effect of carbon support on the activity of monodisperse Co_45_Pt_55_ nanoparticles for oxygen evolution in alkaline media

**DOI:** 10.3389/fchem.2023.1244148

**Published:** 2023-08-07

**Authors:** Stevan Andrić, Jadranka Milikić, Melike Sevim, Diogo M. F. Santos, Biljana Šljukić

**Affiliations:** ^1^ Faculty of Physical Chemistry, University of Belgrade, Belgrade, Serbia; ^2^ Current Affiliation at Center of Microelectronic Technologies, Institute of Chemistry, Technology and Metallurgy, National Institute of the Republic of Serbia, University of Belgrade, Belgrade, Serbia; ^3^ Department of Chemistry, Faculty of Science, Atatürk University, Erzurum, Türkiye; ^4^ Center of Physics and Engineering of Advanced Materials, Laboratory for Physics of Materials and Emerging Technologies, Chemical Engineering Department, Instituto Superior Técnico, Universidade de Lisboa, Lisbon, Portugal

**Keywords:** platinum-cobalt alloy, reduced graphene oxide, mesoporous graphitic carbon nitride, oxygen evolution reaction, alkaline water electrolysis

## Abstract

Oxygen evolution reaction (OER) represents the efficiency-limiting reaction in water electrolyzers, metal-air batteries, and unitized regenerative fuel cells. To achieve high-efficiency OER in alkaline media, we fabricated three novel electrocatalysts by the assembly of as-prepared Co_45_Pt_55_ alloy nanoparticles (NPs) on three different carbon-based support materials: reduced graphene oxide (CoPt/rGO), mesoporous graphitic carbon nitride (CoPt/mpg-CN), and commercial Ketjenblack carbon (CoPt/KB). Voltammetry studies revealed that CoPt/rGO electrocatalyst provided lower OER overpotentials accompanied by higher currents and specific current density values than the other two studied materials. Moreover, CoPt/rGO outperformed commercial CoPt/C electrocatalysts in terms of notably higher specific current densities. Additionally, it was found that CoPt/rGO electrocatalyst activity increases with increasing temperature up to 85°C, as suggested by the increase in the exchange current density. Electrochemical impedance spectroscopy studies of three electrocatalysts in OER revealed similar charge transfer resistance, although CoPt/rGO provided a higher current density. The main issue observed during long-term chronoamperometry and chronopotentiometry studies is the materials’ instability under OER polarization conditions, which is still to be tackled in future work.

## 1 Introduction

The use of fossil fuels as the primary source of energy causes harmful effects on the world and massive environmental problems, particularly global warming (Lee et al., 2013; Santos et al., 2013). According to the European Environmental Agency, the amount of carbon dioxide emitted into the atmosphere is gradually increasing over the last decade due to the use of fossil fuels to meet the increasing energy demand of the world (https://www.eea.europa.eu/publications/carbon-dioxide-emissions-from-europes, n. d.). Therefore, renewable energy sources, such as solar and wind energy, have been studied as alternatives to reduce the world’s fossil fuel dependency (Zendehboudi et al., 2018). However, there are still ongoing technical barriers in front of these green energy sources, such as the discontinuity in the energy supply and the high cost of the first implantation (Zendehboudi et al., 2018). In this regard, electrochemical energy conversion and storage devices have been suggested as complementary ones (Marini et al., 2012; Demir et al., 2019; Du et al., 2019; Santos and Šljukić, 2021). The oxygen evolution reaction (OER) represents a major issue in these systems, specifically in water electrolyzers (Milikić et al., 2021), rechargeable metal-air batteries (Milikic et al., 2022), and unitized regenerative fuel cells (Mladenović et al., 2021; 2022). Equations (1) and (2) describe the OER realized in alkaline (pH 14), acid (pH 0), or neutral media (pH 7), where E_0_ is the equilibrium half-cell potential at standard conditions (Cheng and Jiang, 2015).

$$4{OH}^{-}\;\to \;O_{2}+2H_{2}O+4e^{-}\;E_{0}=0.40\;V\;vs.\;SHE\;\left(alkaline\right)$$ (1)

$$2H_{2}O\;\to \;O_{2}+4H^{+}+4e^{-}\;E_{0}=1.23\;V\;vs.\;SHE\;\left(acidic\right)\;or\;E_{0}=0.82\;V\;vs.\;SHE\;\left(neutral\right)$$ (2)

To achieve the best OER efficiency, with low overpotential and good stability along with low electrocatalyst price, different classes of electrocatalyst materials have been studied. These studies include noble metal-based electrocatalysts, transition metal oxides, oxyhydroxides, perovskite-type oxides, metal phosphides/phosphates/nitrides/borides/sulfides, and carbon-based materials (Tahir et al., 2017). Among them, platinum (Pt) (Pierozynski et al., 2019) and its alloys are found to be the best electrocatalysts for OER (Reier et al., 2012; Milikić et al., 2018; Mladenović et al., 2021). In addition to Pt-based catalysts, ruthenium oxide (RuO_2_) and iridium oxide (IrO_2_) also present excellent catalytic activity for OER in both acidic and alkaline media (Suen et al., 2017). However, they are unstable at high anodic potentials where RuO_2_ is oxidized to RuO_4_ and IrO_2_ is oxidized to IrO_3_ (Suen et al., 2017). Combining Pt with transition metals such as Ni, Fe, or Cu on graphene nanoplatelets was found to notably boost the oxygen electrode kinetics (Mladenović et al., 2022). Besides noble metal-based electrocatalysts, cobalt-based electrodes also show high efficiency towards OER (Cheng and Jiang, 2015; Asadizadeh et al., 2018; Du et al., 2019; Milikić et al., 2021). For instance, Pt-substitution of Co(II, III) oxide led to some improvement of activity towards OER. Specifically, the overpotential to reach the current density of 20 mA cm^−2^ in an alkaline medium decreased by 12 mV (455 mV vs 467 mV) upon 1% Pt-substitution of Co_3_O_4_ (Nellaiappan et al., 2021). This was related to the generation of tetrahedral Co^2+^ after Pt^4+^ substitution where Co^2+^ species is less active towards OER. Furthermore, bimetallic Co-based (CoM, M = Mo, Fe, Mn) coatings showed high efficiency for water splitting in alkaline media (Milikić et al., 2021). Among studied coating, CoMo and Co demonstrated the highest performance for hydrogen evolution reaction (HER) and OER, respectively, reflected in the highest current density with low overpotentials and Tafel slopes. Vanadium nitride (VN) nanowires dual-doped with Co and phosphorus P) (VN-Co-P, VN, VN-Co, and VN-P) presented better OER performance in an alkaline medium due to their higher electronic activity and better physical properties than the undoped VN. This was evidenced by lower overpotential at a current density of 10 mA cm^-2^ of 335, 395, 401, and 458 mV for VN-Co-P, VN-Co, VN-P, and VN, respectively (Yang et al., 2019). Furthermore, boosting electrocatalysis of OER with cost-effective Co, nitrogen (N)-doped carbons prepared by simple carbonization of ionic liquids was proposed within the authors’ recent work (Zdolšek et al., 2022). Elemental Co supported by N atoms was concluded to represent active sites for OER.

On the other hand, the properties of carbon-based electrodes depend on their surface chemistry and microstructure (Yang et al., 2019; Teppor et al., 2020). Graphene has been widely tested for electrochemical sensor applications due to its good electrical and mechanical properties (Compton and Nguyen, 2010). Graphite/graphene oxide (GO)-based materials are attractive electrodes for lithium-ion batteries and electrochemical supercapacitors (Kumar et al., 2012). The oxygen functional groups on the GO surface make it suitable for application as a support material and in composites (Kumar et al., 2012). The graphite-like carbon nitride (g-CN), as a metal-free polymeric photocatalyst, is an inorganic π-conjugated 2D material that can be easily added to the compound’s surface to form core-shell structures (Chen et al., 2014). g-CN has a good photocatalytic activity for hydrogen production under visible light illumination (Hu et al., 2013; Chen et al., 2014). However, the low surface area of g-CN limits its application as a support material for metal nanoparticles (NPs). In this respect, mesoporous g-CN (mpg-CN) has a large surface area and good semiconductor properties leading to enhanced photocatalytic performance (Chen et al., 2014).

This work reports a facile protocol for the synthesis of ultrasmall CoPt alloy NPs and their assembly on three different carbon-based support materials, namely, reduced graphene oxide (rGO), mpg-CN, and commercial Ketjenblack carbon, for the fabrication of efficient electrocatalysts for OER in alkaline solution. These electrocatalysts were thoroughly examined for OER in an alkaline electrolyte solution (1 M KOH) by linear scan voltammetry (LSV), electrochemical impedance spectroscopy (EIS), chronoamperometry (CA), and chronopotentiometry (CP). Finally, their OER activity was compared to that of commercial CoPt/C electrocatalyst.

## 2 Experimental

### 2.1 Materials

Oleylamine (OAm) (>70%), 1-octadecene (ODE, 90%), platinum II) acetylacetonate (Pt (acac)_2_, 99%), cobalt (II) acetylacetonate (Co(acac)_2_, 97%), borane-tert-butylamine (BTB, 97%), hexane (99%), ethanol (99%), acetone (97%), phosphorus pentoxide (P_2_O_5_, 95%), potassium peroxodisulfate (K_2_S_2_O_8_, 95%), potassium permanganate (KMnO_4_, 99%), hydrogen peroxide (H_2_O_2_, 30%), sodium nitrate (NaNO_3_, 99%), sulfuric acid (H_2_SO_4_, 98%), dimethylformamide (DMF, >99%) and Ludox^®^ HS-40 were purchased from Sigma-Aldrich^®^ and used as received. Natural graphite flakes (average particle size: 325 mesh) and guanidine hydrochloride (GndCI, 98%) were purchased from Alfa-Aesar. Ammonium hydrogen difluoride (NH_4_HF_2_, 98.5%) was purchased from Fluka. Commercial Ketjenblack carbon was purchased from Aksa Nobel. Deionized water was distilled using a Milli-Q water purification system.

### 2.2 Instrumentation

All transmission electron microscope (TEM) images were recorded by transmission electron microscope (TEM, Jem-1400 plus electron microscopy, 120 kV). X-ray diffraction (XRD) patterns were recorded on a PANalytical Empyrean diffractometer with Cu-Kα radiation (40 kV, 15 mA, 1.54051 Å) over a 2θ range from 10° to 90° at room temperature. Elemental analysis measurements were carried out by inductively coupled plasma-mass spectroscopy (ICP-MS, Agilent Technologies 7,700) after each sample was completely dissolved in aqua-regia (HCl/HNO_3_: 3/1 vol. ratio).

### 2.3 Synthesis of monodisperse CoPt alloy NPs

Monodisperse Co_45_Pt_55_ alloy NPs were synthesized by using a surfactant-assisted solvothermal method comprising the decomposition and reduction of metal precursors in a solvent-surfactant mixture at a relatively high temperature. In a synthesis of Co_45_Pt_55_ NPs, under an inert atmosphere, 0.2 g of borane-tert-butylamine (BTB), 3 mL of OAm, and 7 mL of ODE were mixed vigorously in a four-necked reactor. Then, a mixture of 0.3 mmol of Pt (acac)_2_ and 0.2 mmol of Co(acac)_2_ was dissolved in 3 mL of OAm at room temperature and injected in the same reactor at 100°C. The reaction mixture was kept at this temperature for an hour and then cooled down to 40°C. The yielded NPs were separated by centrifugation (8,500 rpm, 12 min) after acetone addition (40 mL). This procedure was repeated with ethanol. The obtained NPs were dispersed in hexane for further use.

### 2.4 Preparation of reduced graphene oxide (rGO)

Reduced graphene oxide (rGO) was synthesized by using a well-established two-step wet-chemical procedure starting from natural graphite flakes. In the first step, graphite oxide was synthesized by using the modified Hummers method (Hummers and Offeman, 1958). In the second step, graphite oxide was exfoliated to graphene oxide (GO) in DMF (1 mg mL^-1^) by using an ultrasonic bath, and then the resultant dispersion was refluxed for 6 h. At the end of the refluxing process, GO was reduced to rGO, and floating black rGO sheets were separated from the solution by filtration (Metin et al., 2014).

### 2.5 Preparation of mesoporous graphitic carbon nitride (mpg-CN)

For the synthesis of mpg-CN, we used our established hard-templating method published elsewhere (Aksoy and Metin, 2020). The details of the mpg-CN synthesis procedure and its detailed structural characterization can be found in our recent report (Erdogan et al., 2016).

### 2.6 Assembly of CoPt alloy NPs onto the carbon-based support materials

To use the CoPt alloy NPs as catalysts for OER, they were assembled on rGO, mpg-CN, and commercial Ketjenblack (KB) supports by using the liquid self-assembly method that has been utilized by our group for the synthesis of many electrocatalysts (Şener et al., 2015; Martins et al., 2017; Yılmaz et al., 2019). In a general procedure, 40.0 mg of rGO, mpg-CN, or KB was exfoliated in ethanol by sonicating the solution in an ultrasonic bath for 30 min. Next, 20 mg of the NPs were dispersed in 30 mL of hexane, and this dispersion was mixed with the rGO, mpg-CN, or commercial Ketjenblack dispersion in ethanol. The resultant mixture was sonicated for 2 h to ensure the assembly of the NPs onto the carbon-based support materials. In the final step, the mixture was centrifuged at 7,500 rpm for 10 min and the separated catalyst was washed with ethanol. The alloy composition and the metal contents of the catalyst were determined by ICP-MS.

### 2.7 Electrochemical measurements

All electrochemical measurements were carried out in a 1 M KOH aqueous solution using Ivium V01107 potentiostat/galvanostat in a three-electrode system with graphite rod as the counter electrode, saturated calomel electrode (SCE) as the reference, and CoPt/X (X = rGO, mpg-CN, KB) as the working electrode. All potentials within the paper are given *versus* the reversible hydrogen electrode (RHE).

Working electrodes were prepared by pipetting 10 μL of catalytic ink onto a glassy carbon tip (0.945 cm^2^) and drying it at 100°C for 8 h. Catalytic inks were composed of 5 mg of each electrocatalyst, including CoPt/rGO (6.4 wt% Pt), CoPt/mpg-CN (7.2 wt% Pt), CoPt/KB (1.44 wt% Pt), or commercial Co_25_Pt_75_ (Tanaka Kikinzoku Kogyo Co. Ltd., 46.7 wt% Pt) on carbon, in 125 μL of 2 wt% polyvinylidene difluoride in N-methyl-2-pyrrolidone solution, which were then ultrasonically mixed for 30 min. The final loadings of Pt on the electrode surface are given in Table 1. Accordingly, considering the Co_45_Pt_55_ atomic ratio in the alloy, the carbon-to-catalyst mass ratios were 92.0%, 91.0%, and 98.2% for CoPt/rGO, CoPt/mpg-CN, and CoPt/KB, respectively.

**TABLE 1:** Pt loadings on the prepared electrodes.

Electrocatalyst	Pt loading/mg cm^-2^
CoPt/rGO	0.054
CoPt/mpg-CN	0.084
CoPt/KB	0.009
CoPt/C	0.247

OER studies of the three electrocatalysts were done by LSV in 1 M KOH from the open circuit potential (OCP) to 2 V at 10 mV s^-1^ and different temperatures from 25°C to 85°C, adjusting the temperature by water circulation using a Haake F3 bath. Electrochemical impedance spectroscopy (EIS) measurements were conducted at 25°C in the frequency range of 100 kHz to 0.1 Hz, with 5 mV amplitude, at four different potentials. CA study was carried out at the constant potential of 1.8 V for 1 h, while CP was carried out at the constant current density of 10 mA cm^-2^ for 10 h.

## 3 Results and discussions

### 3.1 Electrocatalysts characterization

**FIGURE 1:**
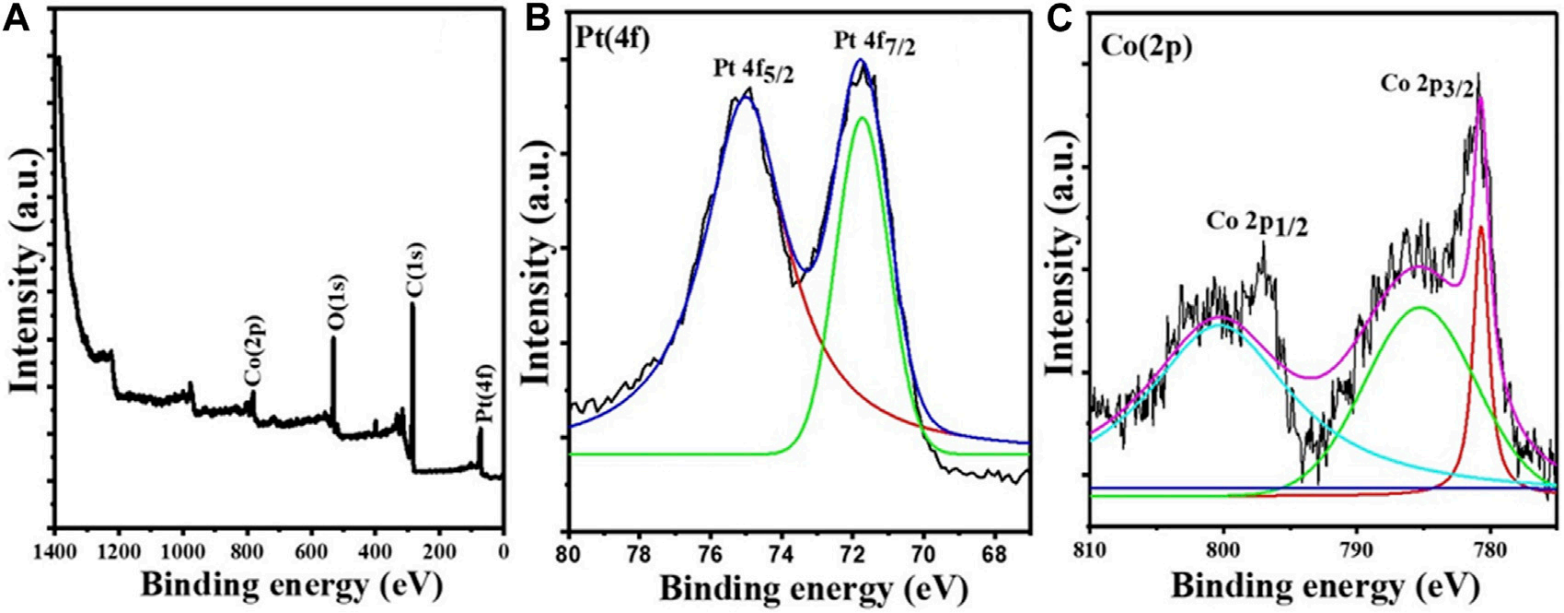
X-ray photoelectron spectroscopy data of the Co_45_Pt_55_ NPs. **(A)** Survey spectrum, **(B)** high-resolution spectrum of the Pt 4f core level, and **(C)** high-resolution spectrum of the Co 2p core level.

Nearly monodisperse CoPt alloy NPs were synthesized by using a new wet-chemical protocol comprising the co-reduction of Co(acac)_2_ and Pt (acac)_2_ precursors with BTB complex in OAm and ODE mixture at 100°C. In the presented recipe, BTB, OAm, and ODE serve as reductant, surfactant, and solvent, respectively. Figure 1 shows the XPS survey and high-resolution spectra for the Pt 4f and Co 2p core levels. The XPS survey spectrum indicates the presence of all expected elements (Pt, Co, O, and C) (Figure 1A). The high-resolution XPS spectrum of the Pt 4f core level (Figure 1B) shows two bands observable at binding energies (BEs) of 71.5 and 74.9 eV, representative of Pt atoms in the metallic state (Alayoglu et al., 2011). In the case of the high-resolution XPS spectrum of the Co 2p core level (Figure 1C), the BEs for Co 2p_1/2_ and Co 2p_3/2_ at 800.6 and 780.1 eV are characteristic of metallic Co.

**FIGURE 2:**
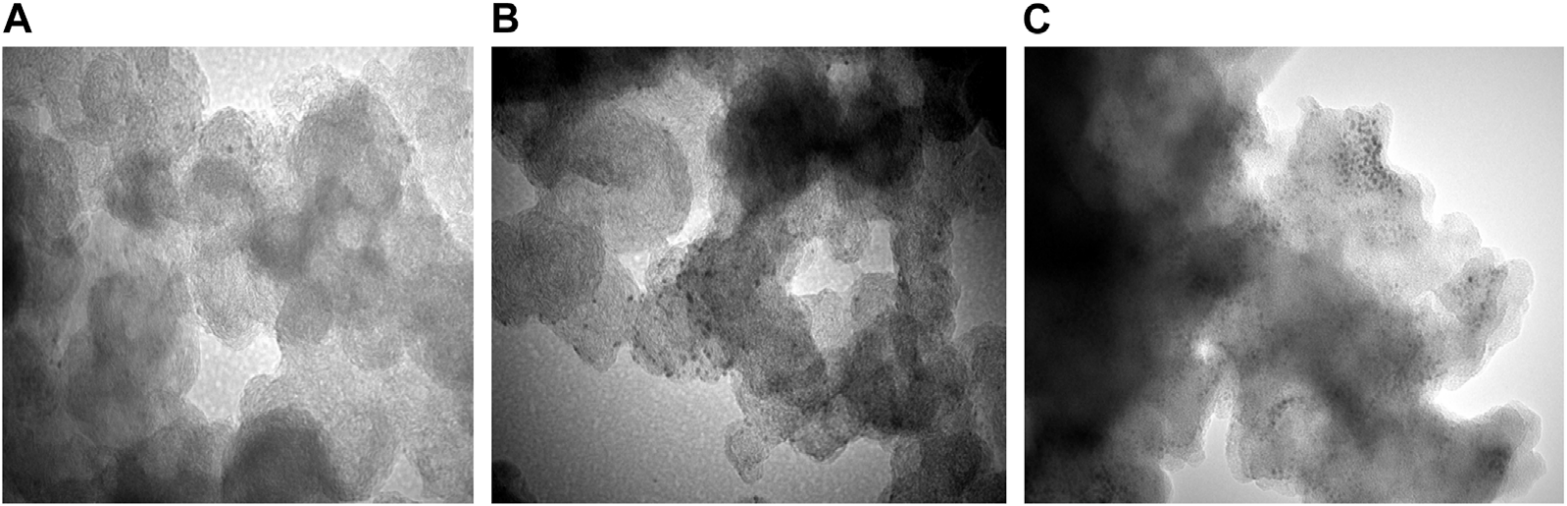
Representative TEM images of **(A)** CoPt/rGO, **(B)** CoPt/mpg-CN, and **(C)** CoPt/KB nanocomposites at a magnification of 200 k.

The colloidal Co_45_Pt_55_ NPs were then assembled on three different carbon-based support materials, namely, rGO, mpg-CN, and commercial KB. The representative TEM images of Co_45_Pt_55_ NPs over these 3 support materials are shown in Figure 2. These images confirm that Co_45_Pt_55_ NPs were successfully assembled on all support materials, but they show different particle dispersion on each support material. CoPt/rGO and CoPt/KB show almost homogenous particle dispersion (Figures 2A, C) while the NPs do not display a completely homogeneous dispersion over mpg-CN (Figure 2B **)**. The observation of such a difference in CoPt NPs dispersion over different carbon-based support materials might be due to the difference in their surface area (BET surface areas; S_rGO_ = 390 m^2^ g^-1^ (Metin et al., 2012), mpg-CN = 182 m^2^ g^-1^ (Erdogan et al., 2016), and S_KB_ = 753 m^2^ g^-1^ (Inoue et al., 2012)). Although there are regions with a higher concentration of NPs in the TEM image of CoPt/mpg-CN nanocomposite, one can say that, overall, CoPt NPs were not agglomerate and preserved their initial particle size and distribution over the three support materials, which allows studying their catalytic activity for the same reaction.

**FIGURE 3:**
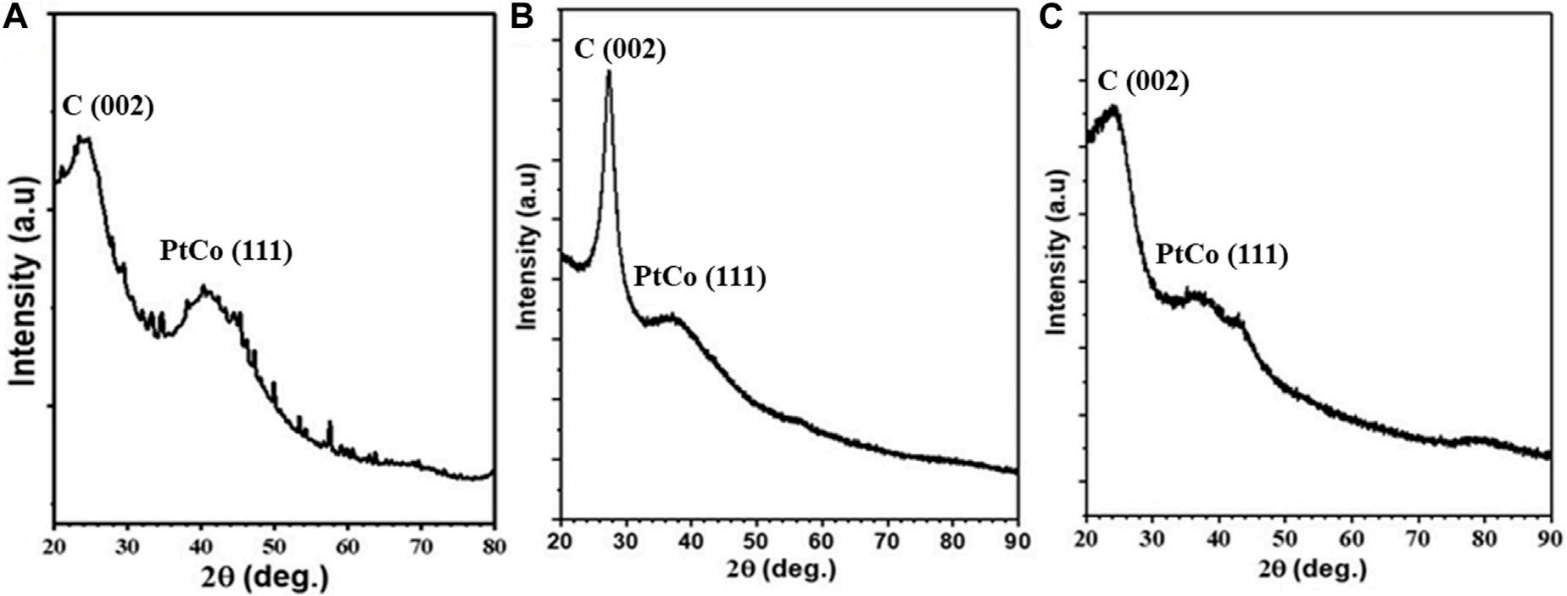
XRD patterns of **(A)** CoPt/rGO, **(B)** CoPt/mpg-CN, and **(C)** CoPt/KB nanocomposites.

To get insight into the crystal structure of Co_45_Pt_55_ alloy NPs, powder XRD analyses were performed on CoPt/rGO, CoPt/mpg-CN, and CoPt/KB nanocomposites. Although all the supported catalysts exhibit mostly amorphous structure, the broad peaks observed at around 2θ = 40° are attributed to the (111) plane of the face-centered cubic crystal phase of CoPt NPs (Figure 3) (Tzitzios et al., 2005; Wang et al., 2018). The broadening of (111) peak is due to the very small particle size of CoPt alloy NPs. Moreover, it should be noted that the (111) peak of CoPt is slightly red-shifted, indicating the solid-solution formation between Pt and Co metals. Additionally, the peak aroused at 2θ = 27.4° for the CoPt/mpg-CN nanocomposite is readily assigned to the (002) plane of mpg-CN and the peaks observed at 2θ = 23° in the XRD patterns of CoPt/rGO and CoPt/KB nanocomposites are attributed to the graphitic layered structure of rGO and commercial carbon.

CV data (not shown) revealed notably higher capacitance in the case of CoPt/rGO than in the case of CoPt/mpg-CN and CoPt/KB. This reflects a higher number of active sites that can participate in the adsorption processes in the O adsorption region and, thus, it is expected to lead to higher OER activity (Eftekhari, 2017). Additionally, the electrocatalytic reactivity of the active sites will depend on their accessibility (as OER is not ideally a surface reaction and it proceeds within a thin layer of ca. 10 nm), as well as on their oxidation state (Milikić et al., 2018).

### 3.2 Oxygen evolution reaction study

**FIGURE 4:**
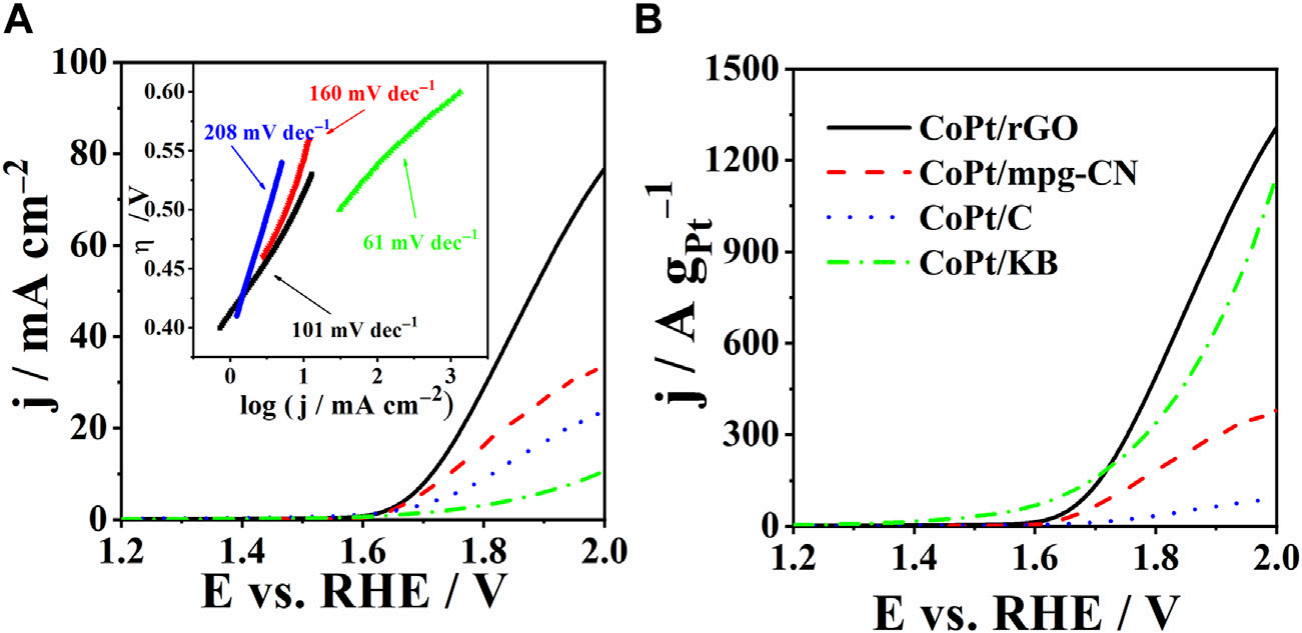
**(A)** Polarization curves of CoPt/rGO, CoPt/mpg-CN, CoPt/KB, and commercial CoPt/C with corresponding Tafel plots in the inset. **(B)** Comparison of specific current densities of the studied electrocatalysts. Measurements were carried out in 1 M KOH at a scan rate of 10 mV s^-1^ and 25°C.

OER activity of CoPt/rGO, CoPt/mpg-CN, and CoPt/KB electrocatalysts was compared by recording polarization curves in 1 M KOH (Figure 4A **)**. The activity was evaluated based on three parameters: onset potential, E_onset_, overpotential necessary to reach a current density of 10 mA cm^-2^, η_10_, and Tafel slope, b. The OER onset potential, defined as the potential at which a current density of 1 mA cm^-2^ is reached (Inoue et al., 2012), was observed to be ca. 10 and 40 mV lower at CoPt/rGO (1.61 V) compared to those of CoPt/mpg-CN (1.62 V) and CoPt/KB (1.64 V), respectively, and ca. 20 mV more positive than that of commercial Pt/C (1.59 V), respectively (Table 2). CoPt/rGO (0.484 V) showed 28 and 308 mV lower overpotential to reach a current density of 10 mA cm^-2^ compared to CoPt/mpg-CN (0.512 V) and CoPt/KB (0.792 V), respectively. Moreover, this value is ca. 100 mV lower than the overpotential value of commercial CoPt/C (0.584 V) and Pt/C (0.576 V) (Mladenović et al., 2021; 2022). Lower overpotential and higher current densities observed in LSVs of the herein-prepared CoPt/rGO point out its superior activity compared to the commercial CoPt/C. This superior performance of CoPt/rGO under OER polarization conditions could be related to the 2D layered structure of rGO that facilitates mass transfer. Moreover, the high surface area of rGO enhances the dispersion of CoPt alloy NPs and facilitates electron transport between the NPs and graphene (Murillo Leo et al., 2017; Meng et al., 2019). As mentioned above, adsorption/desorption is an essential step in the catalysis of water electrolysis, i.e., catalysis of both hydrogen evolution reaction and herein studied OER. rGO was demonstrated to have a key role originating in its unique reactivity related to the O surface groups present in the graphene structure. Namely, it has been shown that rGO acts as H adatoms acceptor facilitating their recombination to form H_2_. H atoms generated by H_2_O discharge on transition metals such as Ni, spill onto the rGO where they are re-combined. At the same time, the electrocatalyst’s surface is continuously cleaned, i.e., free active sites are continuously formed enabling HER/OER to proceed (Chanda et al., 2015).

**TABLE 2:** Comparison of kinetic parameters of OER in alkaline media at 25°C for CoPt/X (X = rGO, mpg-CN, KB, C) and for other noble metal-based electrocatalysts reported in the literature.

Material	E_onset_/V	b/mV dec^−1^	η_10_/V	Source
CoPt/rGO	1.61	101	0.484	This work
CoPt/mpg-CN	1.62	160	0.512	This work
CoPt/KB	1.65	61	0.792	This work
Commercial CoPt/C (46.7 wt% Pt)	1.59	208	0.584	This work
Commercial Pt/C (40 wt% Pt)	-	198	0.576	Mladenović et al. (2021)
CoPt/DTM-C (dif. Co-to-Pt ratios)	-	120–133	0.460–0.380	Wang et al. (2018)
CoPt/C	-	136	0.410	Wang et al. (2018)
sqPtCo/GNPs	-	454	0.690	Mladenović et al. (2023)
sqPtFe/GNPs	-	307	0.582	Mladenović et al. (2023)
sqPtNi/GNPs	-	355	0.654	Mladenović et al. (2023)
PtNi/Mn_2_O_3_	-	174	0.639	Mladenović et al. (2021)
PtNi/Mn_2_O_3_-TiO_2_	-	224	0.858	Mladenović et al. (2021)
PtNi/Mn_2_O_3_-NiO	-	140	0.529	Mladenović et al. (2021)
Pt/GNPs	-	478	0.765	Mladenović et al. (2022)
PtNi/GNPs	-	356	0.652	Mladenović et al. (2022)
PtFe/GNPs	-	280	0.572	Mladenović et al. (2022)
PtCu/GNPs	-	490	0.660	Mladenović et al. (2022)
Ir/C	1.66	164	-	Nguyen and Shim (2018)
Ir_23_Pd_77_/C	1.63	164	-	Nguyen and Shim (2018)
Ir-Pd NHS	-	103	0.398	Zhang et al. (2018)
Ir-Pd nW	-	118	0.464	Zhang et al. (2018)
r-IrO_2_	1.40	-	-	Lee et al. (2012)
r-RuO_2_	1.40	-	-	Lee et al. (2012)

^a^ DTM, diatomite; sq–sequential deposition; GNPs, graphene nanoplatelets; NHS, nanohollow spheres; nW–nanowires; r–rutile.

Furthermore, Tafel slopes were determined from overpotential η vs log current density j plots, Figure 4A (Table 2). The evaluated Tafel slopes of CoPt/KB, CoPt/rGO, CoPt/mpg-CN, and CoPt/C were 61, 101, 160, and 208, mV dec^−1^, respectively, suggesting higher OER rate on CoPt/KB and CoPt/rGO than on CoPt/mpg-CN and commercial CoPt/C. Besides, the OER Tafel slope value for CoPt/rGO was observed to be lower than values reported in the literature, for instance, for OER at commercial Pt/C (198 mV dec^−1^) and PtNi at different transition metal oxide supports (values ranging from 140 to 224 mV dec^−1^) in 0.1 M KOH (Mladenović et al., 2021; Mladenović et al., 2022). Furthermore, PtM (M = Ni, Fe, Cu) on graphene nanoplatelets presented notably higher OER Tafel slope values (280–478 mV dec^−1^). Several Ir_x_Pd_y_/C and Ir/C electrocatalysts were tested for OER in 0.1 M NaOH (Nguyen and Shim, 2018), where Ir_23_Pd_77_/C and Ir/C presented the same value of Tafel slope of 164 mV dec^−1^ that is again higher than Tafel slope value determined for herein tested CoPt/rGO. Ir-Pd nanowires (Zhang et al., 2018) showed a somewhat higher Tafel slope (118 mV dec^−1^) than herein tested CoPt/rGO.

The value of the Tafel slope can further be used to determine the OER mechanism (Eqs. (3)–(7), i.e., the rate-determining step (RDS). Thus, Tafel slopes of ca. 120, 40, and 28 mV dec^−1^ are observed when first (Eq. (3) and (second (Eq. (5)), and third (Eq. (7)) electron transfer is the RDS, respectively (Bandal et al., 2017). In addition, a Tafel slope of 60 mV dec^−1^ can also be observed when the first electron transfer step is followed by the rate-determining chemical transformation (Doyle et al., 2013; Bandal et al., 2017). Shinagawa et al. (2015) thoroughly examined the OER mechanism in alkaline media, where the single-site OER mechanism is assumed. They concluded that the Tafel slope is coverage-dependent. A Tafel slope of 120 mV dec^−1^ was obtained when the adsorbed species formed before the RDS were predominant. On the other hand, a lower Tafel slope was obtained when adsorbed species formed in the first stage of the reaction were predominant. The Tafel slope value evaluated for OER at herein prepared CoPt/rGO electrocatalyst (101 mV dec^−1^) suggests the first electron transfer as the RDS. For comparison, for Pt/C, a Tafel slope of 60 mV dec^−1^ increasing to 120 mV dec^−1^ with increasing potential was reported for OER in 1.0 M KOH (Shinagawa et al., 2015).

$$M+OH^{-}\to MOH+e^{-}$$ (3)

$$MOH+OH^{-}\to MO+H_{2}O_{\left(l\right)}+e^{-}$$ (4)

$$2MO\to 2M+O_{2\left(g\right)}$$ (5)

$$MO+OH^{-}\to MOOH+e^{-}$$ (6)

$$MOOH+OH^{-}\to M+O_{2\left(g\right)}+H_{2}O_{\left(l\right)}+e^{-}$$ (7)

Figure 4B reveals specific current densities of 1402, 1132, 999, and 104 A g^-1^_Pt_ for CoPt/rGO, CoPt/KB, CoPt/mpg-CN, and CoPt/C, respectively, at the potential of 2 V. CoPt/rGO again showed higher activity for OER compared to the commercial CoPt/C electrocatalyst, followed by CoPt/KB, where their specific current densities were ≥10 times higher than the specific current density of commercial CoPt/C. It is worth mentioning that commercial CoPt/C contains 46.7 wt% Pt, whereas CoPt/KB, CoPt/rGO, and CoPt/mpg-CN catalysts contain only 1.4, 6.4, and 7.2 wt% Pt, respectively. Specific current density value at 1.48 V of CoPt/rGO (3.2 A g^-1^_Pt_), was found to be comparable/higher than values reported for rutile IrO_2_ and RuO_2_ NPs, and of the same order of magnitude as that of commercial Ir/C (40 wt% Ir, Premetek, ca. 9 A g^-1^_Ir_ in 0.1 M KOH solution) (Lee et al., 2012).

**FIGURE 5:**
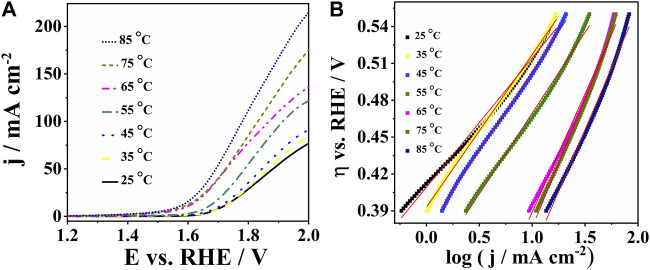
**(A)** Polarization curves of CoPt/rGO in 1 M KOH at different temperatures with **(B)** the corresponding Tafel plots.

Water electrolyzers typically operate at temperatures ranging from 65°C to 90°C. Polarization curves of CoPt/rGO in 1 M KOH illustrate the increase of current densities from 77.2 to 214 mA cm^-2^ with increasing temperature from 25°C to 85°C (Figure 5A **)**. CoPt/mpg-CN electrocatalyst showed some instability with increasing temperatures above 35°C due to vigorous gas bubble evolution. Tafel plots of CoPt/rGO were constructed for all temperatures in the potential range from 1.65 to 1.75 V, allowing the determination of the Tafel slope and exchange current density values (Figure 5B). The exchange current density was observed to increase more than three orders of magnitude, i.e., from 0.13 μA cm^-2^ at 25°C to 170 μA cm^-2^ at 85°C (Table 3), evidencing a strong effect of temperature on OER kinetics (Miles, 2006).

**TABLE 3:** Tafel analysis parameters for OER on CoPt/rGO at different temperatures.

T/°C	Tafel slope/mV dec^−1^	j_0_/μA cm^-2^
**25**	101	0.13
**35**	124	0.70
**45**	130	1.30
**55**	132	2.70
**65**	193	97.0
**75**	209	160
**85**	201	170

**FIGURE 6:**
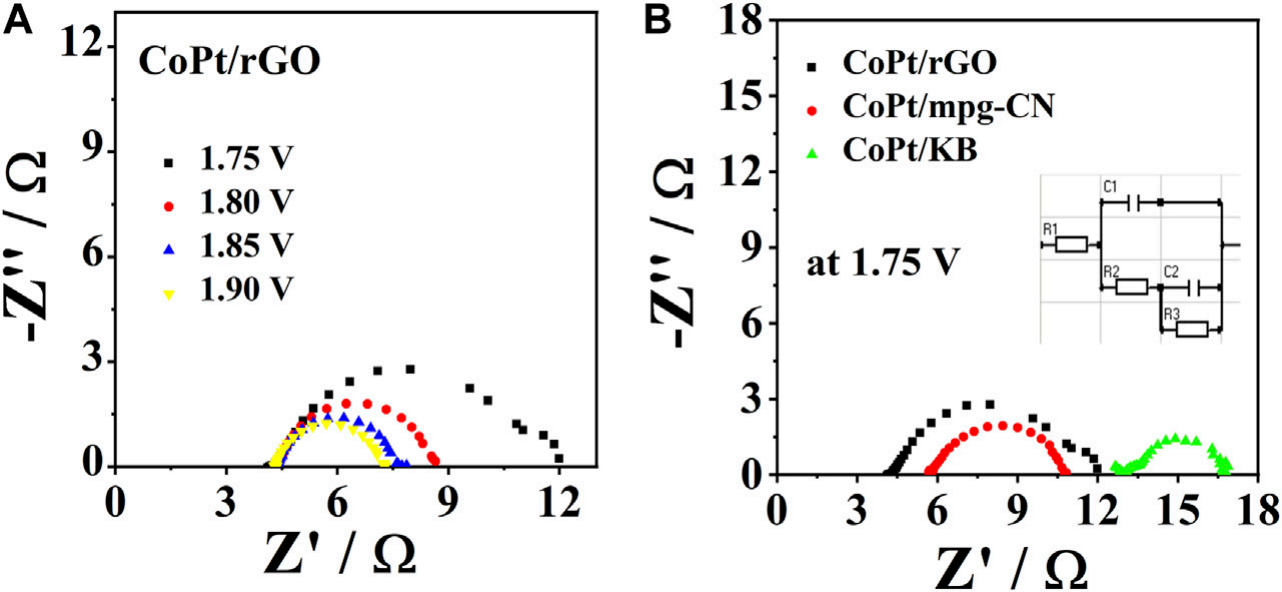
Nyquist plots for OER in 1 M KOH at **(A)** CoPt/rGO at different potentials at 25°C and **(B)** comparison of Nyquist plots of three catalysts recorded at 1.75 V with the equivalent circuit used to fit the impedance data in the insets.

The resistance during OER was assessed by EIS, with the Nyquist plots of three studied electrocatalysts being presented in Figure 6A, B. The electrocatalysts showed the same trend, where resistance values decreased with the increasing applied potential; Figure 6A illustrates the case of CoPt/rGO. The obtained impedance spectra could be best fitted with an equivalent electric circuit having five elements (inset of Figures 6A,B): solution resistance (R_1_), charge transfer resistance at the working electrode (R_2_), mass-transfer resistance of the adsorbed intermediates (pseudo-resistance, R_3_) and capacitance C_1_ and C_2_ related to the double-layer capacitance and the pseudo-capacitance of the working electrode, respectively. Evaluated EIS parameters for OER at CoPt/rGO at four potentials are presented in Table 4 along with the EIS parameters for OER at CoPt/mpg-CN and CoPt/KB electrocatalysts at a potential of 1.75 V. CoPt/rGO, CoPt/mpg-CN, and CoPt/KB showed similar charge transfer resistance values of 1.9, 2.1, and 2.9 Ω, respectively, at 1.75 V (Figure 6C). These low values suggest boosted charge transfer and effective utilization of active material during the electrochemical reaction. The uncompensated solution resistance, R_1_, was slightly lower in the case of CoPt/rGO (4.42 Ω) compared to CoPt/mpg-CN (5.8 Ω) and CoPt/KB (13 Ω); these small variations in solution resistance value originate in small differences in the cell geometry during measurements.

**TABLE 4:** EIS parameters for the OER at CoPt/rGO, CoPt/mpg-CN, and CoPt/KB electrocatalyst in 1 M KOH at 25°C.

Material	E/V	R_1_/Ω	R_2_/Ω	R_3_/Ω	C_1_/mF	C_2_/mF
**CoPt/rGO**	1.70	4.34 ± 0.03	1.6 ± 0.2	4.76 ± 0.05	0.769	1.361
	1.75	4.42 ± 0.02	1.9 ± 0.2	2.2 ± 0.2	0.844	1.301
	1.80	4.398 ± 0.008	1.6 ± 0.9	1.4 ± 0.1	0.870	1.556
	1.85	4.335 ± 0.008	1.5 ± 0.1	1.3 ± 0.1	0.835	1.560
**CoPt/mpg-CN**	1.75	5.8 ± 0.0	2.1 ± 0.4	3.0 ± 0.4	0.37	1.9
**CoPt/KB**	1.75	13 ± 0	2.9 ± 3.44	0.78 ± 9.91	0.52	5.8

**FIGURE 7:**
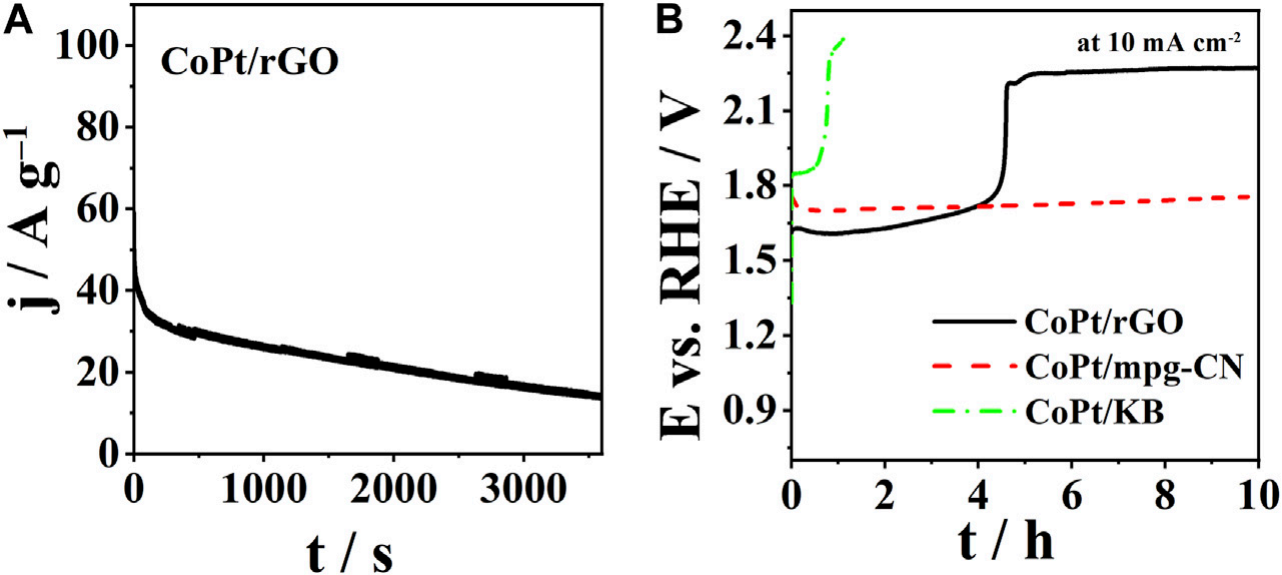
**(A)** CA curves of CoPt/rGO at a potential of 1.8 V, and **(B)** CP curves of three CoPt nanocomposites at a current density of 10 mA cm^-2^.

The chronoamperometric response of CoPt/rGO under OER polarization conditions (constant potential of 1.8 V) is shown in Figure 7A. It could be observed that CoPt/rGO showed OER specific current density of ca. 32 A g^-1^ at 200th s. However (specific) current density at CoPt/rGO was observed to continuously decrease with time. Thereafter, long-term chronopotentiometric measurements were carried out to assess the electrocatalysts’ stability (Figure 7B). CoPt/KB showed instability after less than 1 h. The overpotential to reach a current density of 10 mA cm^-2^ after 10 h was observed to be 0.527 and 1.190 V for CoPt/mpg-CN and CoPt/rGO, respectively. Comparison with values recorded at the beginning of the experiment, reveals the stability of overpotential in the case of CoPt/mpg-CN, but a significant increase in the case of CoPt/rGO. This instability of CoPt/rGO is rather surprising as rGO was reported to stabilize anchored metal NPs through strong electrostatic interactions and thus lead to electrocatalysts’ high long-term stability/durability (Murillo Leo et al., 2017; Meng et al., 2019). Instability might partially come from electrocatalyst’s film deterioration due to intense bubble evolution rather than just NPs detachment from the support. Still, this holds a promise that the stability of CoPt/rGO could be improved and that, along with its high performance for OER catalysis, it could enable its use in water electrolyzers, for instance.

## 4 Conclusion

Three novel electrocatalysts were prepared by the assembly of as-prepared Co_45_Pt_55_ alloy NPs on different carbon-based support materials: reduced graphene oxide (CoPt/rGO), mesoporous graphitic carbon nitride (CoPt/mpg-CN), and commercial carbon black (CoPt/KB). All three electrocatalysts showed high OER efficiency in alkaline media, as confirmed by LSV studies. EIS analysis revealed similar charge transfer properties of the three prepared electrocatalysts. Still, CoPt/rGO was found to be the most efficient for OER among three electrocatalysts, with notably lower Tafel slope and higher current density values than those of commercial CoPt/C. The superior activity of CoPt/rGO becomes even more evident when specific current densities are compared; specific current densities of CoPt/rGO were observed to be ≥10 times higher than that of commercial CoPt/C. However, insight into their stability through short-term chronoamperometric and long-term chronopotentiometric studies revealed that further improvements are vital for enhancing stability.

## Data Availability

The raw data supporting the conclusion of this article will be made available by the authors, without undue reservation.

## References

[B1] Aksoy M. Metin Ö. (2020). Pt nanoparticles supported on mesoporous graphitic carbon nitride as catalysts for hydrolytic dehydrogenation of ammonia borane. ACS Appl. Nano Mater. 3, 6836–6846. 10.1021/acsanm.0c01208

[B2] Alayoglu S. Beaumont S. K. Zheng F. Pushkarev V. V. Zheng H. Iablokov V. (2011). CO_2_ hydrogenation studies on Co and CoPt bimetallic nanoparticles under reaction conditions using TEM, XPS and NEXAFS. Top. Catal. 54, 778–785. 10.1007/s11244-011-9695-9

[B3] Asadizadeh S. Amirnasr M. Meghdadi S. Fadaei Tirani F. Schenk K. (2018). Facile synthesis of Co_3_O_4_ nanoparticles from a novel tetranuclear cobalt(III) complex. Application as efficient electrocatalyst for oxygen evolution reaction in alkaline media. Int. J. Hydrogen Energy 43, 4922–4931. 10.1016/j.ijhydene.2018.01.104

[B4] Bandal H. A. Jadhav A. R. Tamboli A. H. Kim H. (2017). Bimetallic iron cobalt oxide self-supported on Ni-Foam: An efficient bifunctional electrocatalyst for oxygen and hydrogen evolution reaction. Electrochimica Acta 249, 253–262. 10.1016/j.electacta.2017.07.178

[B5] Chanda D. Hnát J. Dobrota A. S. Pašti I. A. Paidar M. Bouzek K. (2015). The effect of surface modification by reduced graphene oxide on the electrocatalytic activity of nickel towards the hydrogen evolution reaction. Phys. Chem. Chem. Phys. 17, 26864–26874. 10.1039/C5CP04238K 26399740

[B6] Chen D. Wang K. Xiang D. Zong R. Yao W. Zhu Y. (2014). Significantly enhancement of photocatalytic performances via core-shell structure of ZnO@mpg-C_3_N_4_ . Appl. Catal. B Environ. 147, 554–561. 10.1016/j.apcatb.2013.09.039

[B7] Cheng Y. Jiang S. P. (2015). Advances in electrocatalysts for oxygen evolution reaction of water electrolysis-from metal oxides to carbon nanotubes. Prog. Nat. Sci. Mater. Int. 25, 545–553. 10.1016/j.pnsc.2015.11.008

[B8] Compton O. C. Nguyen S. T. (2010). Graphene oxide, highly reduced graphene oxide, and graphene: Versatile building blocks for carbon-based materials. Small 6, 711–723. 10.1002/smll.200901934 20225186

[B9] Demir E. Akbayrak S. Önal A. M. Özkar S. (2019). Ceria supported ruthenium(0) nanoparticles: Highly efficient catalysts in oxygen evolution reaction. J. Colloid Interface Sci. 534, 704–710. 10.1016/j.jcis.2018.09.075 30268935

[B10] Doyle R. L. Godwin I. J. Brandon M. P. Lyons M. E. G. (2013). Redox and electrochemical water splitting catalytic properties of hydrated metal oxide modified electrodes. Phys. Chem. Chem. Phys. 15, 13737–13783. 10.1039/c3cp51213d 23652494

[B11] Du Y. Qu H. Liu Y. Han Y. Wang L. Dong B. (2019). Bimetallic CoFeP hollow microspheres as highly efficient bifunctional electrocatalysts for overall water splitting in alkaline media. Appl. Surf. Sci. 465, 816–823. 10.1016/j.apsusc.2018.09.231

[B12] Eftekhari A. (2017). From pseudocapacitive redox to intermediary adsorption in oxygen evolution reaction. Mater. Today Chem. 4, 117–132. 10.1016/j.mtchem.2017.03.003

[B13] Erdogan D. A. Sevim M. Kısa E. Emiroglu D. B. Karatok M. Vovk E. I. (2016). Photocatalytic activity of mesoporous graphitic carbon nitride (mpg-C_3_N_4_) towards organic chromophores under UV and VIS light illumination. Top. Catal. 59, 1305–1318. 10.1007/s11244-016-0654-3

[B14] Hu M. Shan G. Fu Y. Wang C. Zhu L. Chang C. (2013). Photodegradation of bisphenol A by highly stable palladium-doped mesoporous graphite carbon nitride (Pd/mpg-C_3_N_4_) under simulated solar light irradiation. Appl. Catal. B Environ. 142–143, 553–560. 10.1016/j.apcatb.2013.05.044

[B15] Hummers W. S. Offeman R. E. (1958). Preparation of graphitic oxide. J. Am. Chem. Soc. 80, 1339. 10.1021/ja01539a017

[B16] Inoue H. Hosoya K. Kannari N. Ozaki J. I. (2012). Influence of heat-treatment of Ketjen Black on the oxygen reduction reaction of Pt/C catalysts. J. Power Sources 220, 173–179. 10.1016/j.jpowsour.2012.07.101

[B17] Kumar N. A. Choi H. J. Shin Y. R. Chang D. W. Dai L. Baek J. B. (2012). Polyaniline-grafted reduced graphene oxide for efficient electrochemical supercapacitors. ACS Nano 6, 1715–1723. 10.1021/nn204688c 22276770

[B18] Lee J. Jeong B. Ocon J. D. (2013). Oxygen electrocatalysis in chemical energy conversion and storage technologies. Curr. Appl. Phys. 13, 309–321. 10.1016/j.cap.2012.08.008

[B19] Lee Y. Suntivich J. May K. J. Perry E. E. Shao-Horn Y. (2012). Synthesis and activities of rutile IrO_2_ and RuO_2_ nanoparticles for oxygen evolution in acid and alkaline solutions. J. Phys. Chem. Lett. 3, 399–404. 10.1021/jz2016507 26285858

[B20] Marini S. Salvi P. Nelli P. Pesenti R. Villa M. Berrettoni M. (2012). Advanced alkaline water electrolysis. Electrochimica Acta 82, 384–391. 10.1016/j.electacta.2012.05.011

[B21] Martins M. Šljukić B. Metin Ö. Sevim M. Sequeira C. A. C. Şener T. (2017). Bimetallic PdM (M = Fe, Ag, Au) alloy nanoparticles assembled on reduced graphene oxide as catalysts for direct borohydride fuel cells. J. Alloys Compd. 718, 204–214. 10.1016/J.JALLCOM.2017.05.058

[B22] Meng H. B. Zhang X. F. Pu Y. L. Chen X. L. Feng J. J. Han D. M. (2019). One-pot solvothermal synthesis of reduced graphene oxide-supported uniform PtCo nanocrystals for efficient and robust electrocatalysis. J. Colloid Interface Sci. 543, 17–24. 10.1016/j.jcis.2019.01.110 30772535

[B23] Metin Ö. Aydoǧan Ş. Meral K. (2014). A new route for the synthesis of graphene oxide-Fe_3_O_4_ (GO-Fe_3_O_4_) nanocomposites and their Schottky diode applications. J. Alloys Compd. 585, 681–688. 10.1016/j.jallcom.2013.09.159

[B24] Metin Ö. Kayhan E. Özkar S. Schneider J. J. (2012). Palladium nanoparticles supported on chemically derived graphene: An efficient and reusable catalyst for the dehydrogenation of ammonia borane. Int. J. Hydrogen Energy 37, 8161–8169. 10.1016/j.ijhydene.2012.02.128

[B25] Miles M. H. Kissel G. Lu P. W. T. Srinivasan S. (2006). Effect of temperature on electrode kinetic parameters for hydrogen and oxygen evolution reactions on nickel electrodes in alkaline solutions. J. Electrochem. Soc. 123, 332–336. 10.1149/1.2132820

[B26] Milikić J. Balčiūnaitė A. Sukackienė Z. Mladenović D. Santos D. M. F. Tamašauskaitė-Tamašiūnaitė L. (2021). Bimetallic co-based (Com, m = mo, fe, mn) coatings for high-efficiency water splitting. Materials 14, 92–15. 10.3390/ma14010092 PMC7795325 33379230

[B27] Milikic J. M. Cfuentes R. O. Tasca J. E. Santos D. M. F. Šljukic B. Figueiredo F. M. L. (2022). Nickel-doped ceria bifunctional electrocatalysts for oxygen reduction and evolution in alkaline media. Batter. (Basel) 8, 100. 10.3390/BATTERIES8080100

[B28] Milikić J. Vasić M. Amaral L. Cvjetićanin N. Jugović D. Hercigonja R. (2018). NiA and NiX zeolites as bifunctional electrocatalysts for water splitting in alkaline media. Int. J. Hydrogen Energy 43, 18977–18991. 10.1016/j.ijhydene.2018.08.063

[B29] Mladenović D. Daş E. Santos D. M. F. Bayrakçeken Yurtcan A. Šljukić B. (2023). Highly efficient oxygen electrode obtained by sequential deposition of transition metal-platinum alloys on graphene nanoplatelets. Materials 16, 3388. 10.3390/ma16093388 37176270 PMC10179827

[B30] Mladenović D. Daş E. Santos D. M. F. Yurtcan A. B. Miljanić Š. Šljukić B. (2022). Boosting oxygen electrode kinetics by addition of cost-effective transition metals (Ni, Fe, Cu) to platinum on graphene nanoplatelets. J. Alloys Compd. 905, 164156. 10.1016/J.JALLCOM.2022.164156

[B31] Mladenović D. Santos D. M. F. Bozkurt G. Soylu G. S. P. Yurtcan A. B. Miljanić Š. (2021). Tailoring metal-oxide-supported PtNi as bifunctional catalysts of superior activity and stability for unitised regenerative fuel cell applications. Electrochem. Commun. 124, 106963. 10.1016/j.elecom.2021.106963

[B32] Murillo Leo I. Soto E. Vaquero F. Mota N. Navarro R. M. Fierro J. L. G. (2017). Influence of the reduction of graphene oxide (rGO) on the structure and photoactivity of CdS-rGO hybrid systems. Int. J. Hydrogen Energy 42, 13691–13703. 10.1016/j.ijhydene.2016.11.154

[B33] Nellaiappan S. Jhariya N. Irusta S. Singhal A. (2021). Platinum substituted Cobalt(II, III) Oxide: Interplay of tetrahedral Co(II) sites towards electrochemical oxygen evolution activity. Electrochimica Acta 365, 137234. 10.1016/J.ELECTACTA.2020.137234

[B34] Nguyen A. T. N. Shim J. H. (2018). Facile one-step synthesis of Ir-Pd bimetallic alloy networks as efficient bifunctional catalysts for oxygen reduction and oxygen evolution reactions. J. Electroanal. Chem. 827, 120–127. 10.1016/j.jelechem.2018.09.012

[B35] Pierozynski B. Mikolajczyk T. Luba M. Zolfaghari A. (2019). Kinetics of oxygen evolution reaction on nickel foam and platinum-modified nickel foam materials in alkaline solution. J. Electroanal. Chem. 847, 113194. 10.1016/J.JELECHEM.2019.113194

[B36] Reier T. Oezaslan M. Strasser P. (2012). Electrocatalytic oxygen evolution reaction (OER) on Ru, Ir, and pt catalysts: A comparative study of nanoparticles and bulk materials. ACS Catal. 2, 1765–1772. 10.1021/cs3003098

[B37] Santos D. M. F. Šljukić B. (2021). Advanced materials for electrochemical energy conversion and storage devices. Materials 14, 7711–7716. 10.3390/ma14247711 34947306 PMC8706487

[B38] Santos D. M. F. Šljukić B. Sequeira C. A. C. Macciò D. Saccone A. Figueiredo J. L. (2013). Electrocatalytic approach for the efficiency increase of electrolytic hydrogen production: Proof-of-concept using platinum-dysprosium alloys. Energy 50, 486–492. 10.1016/j.energy.2012.11.003

[B39] Şener T. Kayhan E. Sevim M. Metin Ö. (2015). Monodisperse CoFe_2_O_4_ nanoparticles supported on Vulcan XC-72: High performance electrode materials for lithium-air and lithium-ion batteries. J. Power Sources 288, 36–41. 10.1016/j.jpowsour.2015.04.120

[B40] Shinagawa T. Garcia-Esparza A. Takanabe K. (2015). Insight on Tafel slopes from a microkinetic analysis of aqueous electrocatalysis for energy conversion. Sci. Rep. 5, 13801. 10.1038/srep13801 26348156 PMC4642571

[B41] Suen N.-T. Hung S.-F. Quan Q. Zhang N. Xu Y.-J. Chen H. M. (2017). Electrocatalysis for the oxygen evolution reaction: Recent development and future perspectives. Chem. Soc. Rev. 46, 337–365. 10.1039/c6cs00328a 28083578

[B42] Tahir M. Pan L. Idrees F. Zhang X. Wang L. Zou J. J. (2017). Electrocatalytic oxygen evolution reaction for energy conversion and storage: A comprehensive review. Nano Energy 37, 136–157. 10.1016/j.nanoen.2017.05.022

[B43] Teppor P. Jäger R. Paalo M. Palm R. Volobujeva O. Härk E. (2020). Peat-derived carbon-based non-platinum group metal type catalyst for oxygen reduction and evolution reactions. Electrochem. Commun. 113, 106700. 10.1016/j.elecom.2020.106700

[B44] Tzitzios V. Niarchos D. Gjoka M. Boukos N. Petridis D. (2005). Synthesis and characterization of 3D CoPt nanostructures. J. Am. Chem. Soc. 127 (40), 13756–13757. 10.1021/ja053044m 16201773

[B45] Wang K. Tang Z. Wu W. Xi P. Liu D. Ding Z. (2018). Nanocomposites CoPt-x/Diatomite-C as oxygen reversible electrocatalysts for zinc-air batteries: Diatomite boosted the catalytic activity and durability. Electrochimica Acta 284, 119–127. 10.1016/j.electacta.2018.07.154

[B46] Yang H. Hu Y. Huang D. Xiong T. Li M. Balogun M. S. (2019). Efficient hydrogen and oxygen evolution electrocatalysis by cobalt and phosphorus dual-doped vanadium nitride nanowires. Mater. Today Chem. 11, 1–7. 10.1016/j.mtchem.2018.10.004

[B47] Yılmaz M. S. Kaplan B. Y. Gürsel S. A. Metin Ö. (2019). Binary CuPt alloy nanoparticles assembled on reduced graphene oxide-carbon black hybrid as efficient and cost-effective electrocatalyst for PEMFC. Int. J. Hydrogen Energy 44, 14184–14192. 10.1016/j.ijhydene.2018.11.228

[B48] Zdolšek N. Vujković M. Metin Ö. Brković S. Jocić A. Dimitrijević A. (2022). Boosting electrocatalysis of oxygen reduction and evolution reactions with cost-effective cobalt and nitrogen-doped carbons prepared by simple carbonization of ionic liquids. Int. J. Hydrogen Energy 47, 14847–14858. 10.1016/J.IJHYDENE.2022.02.225

[B49] Zendehboudi A. Baseer M. A. Saidur R. (2018). Application of support vector machine models for forecasting solar and wind energy resources: A review. J. Clean. Prod. 199, 272–285. 10.1016/j.jclepro.2018.07.164

[B50] Zhang T. Liao S. Dai L. Yu J. Zhu W. Zhang Y. (2018). Ir-Pd nanoalloys with enhanced surface-microstructure-sensitive catalytic activity for oxygen evolution reaction in acidic and alkaline media. Sci. China Mater. 61, 926–938. 10.1007/s40843-017-9187-1

